# Progress on lipid extraction from wet algal biomass for biodiesel production

**DOI:** 10.1111/1751-7915.12360

**Published:** 2016-05-19

**Authors:** Forough Ghasemi Naghdi, Lina M González González, William Chan, Peer M Schenk

**Affiliations:** ^1^ Algae Biotechnology LaboratorySchool of Agriculture and Food SciencesThe University of QueenslandBrisbaneQLD4072Australia

## Abstract

Lipid recovery and purification from microalgal cells continues to be a significant bottleneck in biodiesel production due to high costs involved and a high energy demand. Therefore, there is a considerable necessity to develop an extraction method which meets the essential requirements of being safe, cost‐effective, robust, efficient, selective, environmentally friendly, feasible for large‐scale production and free of product contamination. The use of wet concentrated algal biomass as a feedstock for oil extraction is especially desirable as it would avoid the requirement for further concentration and/or drying. This would save considerable costs and circumvent at least two lengthy processes during algae‐based oil production. This article provides an overview on recent progress that has been made on the extraction of lipids from wet algal biomass. The biggest contributing factors appear to be the composition of algal cell walls, pre‐treatments of biomass and the use of solvents (e.g. a solvent mixture or solvent‐free lipid extraction). We compare recently developed wet extraction processes for oleaginous microalgae and make recommendations towards future research to improve lipid extraction from wet algal biomass.

## Introduction

**Figure 1:**
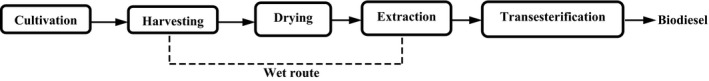
A pipeline model for the production of biodiesel from microalgae. Microalgae are cultivated by providing light, nutrients (especially nitrogen and phosphate), carbon dioxide and water in an open or closed bioreactor. Once there is concentrated biomass, it is harvested and dewatered by sedimentation, flocculation, filtration, centrifugation, flotation or electrophoresis techniques. Lipid extraction is performed after cell disruption. A ‘wet route’ would eliminate the drying process while lowering the production costs. The final step for biodiesel production is algal oil transesterification.

Microalgal feedstocks have been considered a suitable alternative to traditional oil‐bearing crops as a source of biodiesel production due to simple cultivation systems, reduced pressure on competing arable land and natural resources, and the potential to meet current demands of global biofuel mandates (Singh and Dhar, 2011). The production of biodiesel from microalgae consists of extracting cytosolic lipid bodies that contain large amounts of triacylglycerides (TAG) and can be further refined into biodiesel *via* transesterification (Chisti, 2007; Hu *et al*., 2008). Table 1 provides an overview of microalgal strains that have been assessed for TAG production as potential feedstock for biodiesel or other algal oil‐based products. However, due to the presence of thick and robust algal cell walls, releasing the lipids has become a major drawback for manufacturing biodiesel, as techniques that are currently in place are derived from expensive conventional methods for oil‐bearing terrestrial crops. These include concentrating and dewatering the microalgae before undergoing extraction, which not only are energy intensive and ineffective but also toxic due to organic solvents, deeming them unfeasible on the industrial scale (Uduman *et al*., 2010; de Boer *et al*., 2012; Lee *et al*., 2012; Passell *et al*., 2013; Torres *et al*., 2013). To mitigate this, lipid extraction from wet algal biomass has been proposed as an ideal solution, disrupting the algal cells in solution and eliminating the costs and impacts that are associated above (shown in Fig. 1). In this paper, we review recent progress made towards developing wet lipid extraction techniques and their constraints.

**Table 1:** Examples of microalgal strains being evaluated for TAG production

Strain	Lipid content (% of DCW)	Lipid productivity (mg/L/d)	Reference
*Chaetoceros calcitrans* CS 178	40	18	Rodolfi *et al*. (2009)
*Chaetoceros muelleri* F&M‐M43	34	22	Rodolfi *et al*. (2009)
*Chlorella protothecoides*	43–46	1881–1840	Cheng *et al*. (2009)
*Chlorella protothecoides*	55	932	Xu *et al*. (2006)
*Chlorella sorokiniana* IAM‐212	19	45	Rodolfi *et al*. (2009)
*Chlorella vulgaris*	21	254	Liang *et al*. (2009)
*Chlorella vulgaris*	5.1	180	Gouveia and Oliveira (2009)
*Chlorella zofingiensis*	51	354	Liu *et al*. (2011)
*Chlorococcum* sp. UMACC 112	19	54	Rodolfi *et al*. (2009)
*Dunaliella tertiolecta*	17	120	Gouveia and Oliveira (2009)
*Isochrysis* sp. F&M‐M28	22	38	Rodolfi *et al*. (2009)
*Monodus subterraneus* UTEX 151	16	30	Rodolfi *et al*. (2009)
*Nannochloropsis oculata*	23–30	84–142	Chiu *et al*. (2009)
*Nannochloropsis* sp.	35–48	385–413	Pal *et al*. (2011)
*Nannochloropsis* sp.	29	90	Gouveia and Oliveira (2009)
*Neochloris oleoabundans*	29	90	Gouveia and Oliveira (2009)
*Pavlova lutheri* CS 182	36	50	Rodolfi *et al*. (2009)
*Scenedesmus obliquus*	39	79	Ho *et al*. (2010)
*Scenedesmus obliquus*	18	90	Gouveia and Oliveira (2009)
*Scenedesmus quadricauda*	18	35	Rodolfi *et al*. (2009)
*Spirulina maxima*	4	210	Gouveia and Oliveira (2009)
*Tetraselmis* sp. F&M‐M34	15	43	Rodolfi *et al*. (2009)

DCW, dry cell weight.

## Recent progress in lipid extraction from wet algal biomass

By definition, lipid extraction from wet algal biomass consists of disrupting/damaging the algal cell walls in the solution in which the microalgae were cultivated in. These techniques can be grouped into organic solvent‐based and solvent‐free approaches. Considering solvent‐based lipid extraction techniques on wet biomass, water plays a barrier role between the intracellular lipids and non‐ploar organic solvents. Increasing the polarity of the solvent can remove this obstacle to a great extent. Safety and environmental issues are the main limitations of organic solvent‐based techniques, so a new direction of research focuses on developing organic solvent‐free approaches which are not only safe and environmental friendly but also minimizing the need to separate contaminants from the extracted lipid product. The most desirable solvent‐free techniques are the ones which can be implemented on a diverse range of algal strains with low energy consumption and negligible initial set‐up costs for infrastructure. As discussed below, promising approaches come from microalgal cell rupturing techniques that free up lipid bodies which subsequently need to be recovered. Although still immature, solvent‐free extraction is seen to be a promising technique for the industrial production of primary extracted lipids. Similar to the vegetable industry, the feasibility of secondary extraction of remaining cellular lipids from partially defatted algae by using organic solvents needs to be assessed.

## Pre‐treatments of biomass

For microalgae lipid extraction, the cells have to be disrupted properly, irrespective if an organic solvent is to be used or not. Pre‐treatments can be applied on the biomass to enhance lipid recovery efficiency through breaking or weakening the microalgal cell walls which in turn facilitates easier cellular lipid extraction (Cooney *et al*., 2009; Yoo *et al*., 2012). There are different methods which can be applied as pre‐treatments; including mechanical, chemical, physical and biological approaches. Some examples are pressing, bead milling, electroporation, homogenization and osmotic shock (mechanical), cell lysing with acid/base (chemical), lyophilization, sonication, microwave, thermal (physical) and enzymatic polysaccharide and/or protein degradation (biological) (Kita *et al*., 2010; Mercer and Armenta, 2011; Kim *et al*., 2013). In mechanical pre‐treatments (compared with chemical and biological pre‐treatments), the risk of degradation or degeneration of the target compound is considered reduced (Greenwell *et al*., 2009; Kim *et al*., 2013). Although almost all of these pre‐treatments are used in conjunction with solvent techniques for the recovery of extracted lipids, less solvent can be used compared with untreated biomass (Mercer and Armenta, 2011).

## Solvent‐based techniques

### Use of solvent mixtures

As mentioned before, solvents still play a main role in both extraction and recovery of microalgal lipids. Suitable solvents should be chosen as per the target compound polarity. The main microalgal lipid material for biodiesel production are TAGs, which are non‐polar and hence, more soluble in non‐polar organic solvents (Levine *et al*., 2010; Chen *et al*., 2012). However, a successful extraction solvent is one which can fully penetrate the biomass, making physical contact with the targeted lipid material and subsequently dissolve it completely (Mercer and Armenta, 2011). The lipid extraction/recovery can be enhanced through increasing the polarity of the solvent by mixing the polar and non‐polar solvents. This is due to the ability of the polar solvents to release the lipids from their protein–lipid complexes which facilitates their dissolving in the non‐polar solvent (Ryckebosch *et al*., 2012). This effect is even higher when the lipid extraction/recovery is to be performed on wet biomass, as the polar solvent can penetrate the water layer and make the lipids available for non‐polar solvent solvation (Yoo *et al*., 2012). We recently reported that not only the total lipid recovery can be increased through utilizing a solvent mixture but also the total FAME recovery can be increased when using a mixture of polar and non‐polar solvents (Ghasemi Naghdi *et al*., 2014). Utilizing a solvent mixture of hexane and ethanol at a 3:1 ratio, respectively, through a Soxhlet system, when applied on *Tetraselmis* sp. significantly enhanced FAME recovery by 50%.

### Microwave‐assisted extraction

Microwave‐assisted extractions (MAE) were first established in the mid 1980s as a means to obtain lipids and pesticides from seeds, foods, feeds and soil (Ganzler *et al*., 1986). When applied to microalgal cultures, microwave technology has been proven to be not only relatively safe, rapid and economical but also reduced the cost associated with dewatering and extracting of dry algal biomass (Lee *et al*., 2010). Typically, the contact between a dielectric or polar material (e.g. water) and a rapidly oscillating electric field (produced by microwaves) generates heat due to frictional forces arising from inter‐ and intra‐molecular movements. As heat is produced, water vapour begins to form within the cell, eventually rupturing it, leading to an electroporation effect which further opens up the cell membrane and releasing the intracellular contents (Amarni and Kadi, 2010). Šoštarič *et al*. (2012) previously reported that microwaves, when used in conjunction with other mechanical extraction methods such as sonication, have yielded higher levels of lipid in *Chlorella vulgaris*, while Refaat *et al*. (2008) indicated that microwave irradiation can also assist in the transesterification process post extraction by substituting conventional heating. When implementing MAE, considerations regarding the time, temperature, dielectric properties of the process mixture, the solid–liquid ratio, and the type and concentration of the solvent should be taken into account (Eskilsson and Björklund, 2000). For example, Balasubramanian *et al*. (2011) demonstrated that higher temperatures at longer times resulted in higher oil extraction efficiency when compared with standard methods such as the Soxhlet extraction; however, the presence of TAGs is highest at elevated temperatures accompanied by shorter times. However, if the target compounds and/or the solvent(s) are non‐polar or volatile, the efficiency of MAE can be hindered dramatically (Wang and Weller, 2006). Currently, MAE is evaluated to be a cost‐effective method for wet lipid extraction from microalgae based on short reaction times and the extraction of high‐quality lipids; however, when scaling up commercially, maintenance cost is viewed as a limiting factor.

### Ultrasound‐assisted extraction

Ultrasound‐assisted extraction (UAE) eliminates the issues associated with conventional mechanical disruption, and is advantageous due to low set‐up costs, fast operational time and high purity of the final product. In the presence of liquid cultures, UAE can rupture the cells *via* cavitation which produces microbubbles around the cell as a result of an ultrasonic wave. The eventual collapse of these bubbles emits a shockwave which shatters the cell wall, hence releasing the intracellular contents (Suslick and Flannigan, 2008; Harun *et al*., 2010). Metherel *et al*. (2009) showed that an increase in amplitude of the exposure time can result in higher lipid recoveries, with further enhancement using a mixture of polar and non‐polar solvents, while Vinatoru *et al*. (1997) demonstrated that UAE can not only reduce extraction time but also facilitate the absorption of cell contents into the solvent through mass transfer and penetration of the solvent into the cell. UAE extraction using ethanol as the only solvent may improve environmental safety. In addition, UAE can also be advantageous to MAE as it can be conducted under low temperatures, reducing thermal denaturation of essential biomolecules (Ranjith Kumar *et al*., 2015). However, if the microalgal cultures are subjected to prolonged exposure to sonication, this can lead to the generation of free radicals that can deteriorate the quality of the lipids through oxidation (Chemat *et al*., 2004). Nevertheless, oxidation can be limited by utilizing non‐polar organic solvents which are not susceptible to peroxide formation, such as hexane. Metherel *et al*. (2009) reported that the ideal ratio of extraction solvents for UAE in flaxseed is 2:1 chloroform:methanol and 3:2 hexane:isopropanol, reducing lipid oxidation and resulting in higher yields.

### Hydrothermal liquefaction

Hydrothermal liquefaction (HTL) is a thermochemical conversion technique that processes the whole microalgal biomass by applying medium to subcritical temperature (below 374°C) and high pressure (10–25 MPa) (Garcia Alba *et al*., 2011; Barreiro *et al*., 2013). Under hydrothermal conditions, fatty acids and hydrogenated compounds (‘biocrude’) are produced from lipids (Biller *et al*., 2011). As substantial equipment and running costs are associated with this technology, economical feasibility and scalability need to be considered. While these still need to be demonstrated, HTL reportedly achieved an energy recovery from biomass to fuel up to 80% (Toor *et al*., 2011). As HTL can be used on wet algal biomass with a water content as high as 80–95%, it was also claimed to require less than 5% of the energy costs otherwise needed for complete drying (Garcia Alba *et al*., 2011). Toor *et al*. (2013) reported a biocrude yield (lipid conversion to free fatty acids) around 34–38% for *Spirulina platensis* after HTL at 310°C and 115 bar and 34–46% for *Nannochloropsis salina* at 350°C and 175 bar. A biocrude yield of 27% and 47% from HTL of *Scenedesmus* (350°C) and *Chlorella* (300°C), respectively, was reported by Biller *et al*. (2012). These conversion rates are very low when compared with other feedstocks, e.g. for soybean where, with the right catalyst, more than 90% of the lipids can be converted to fatty acids. The use of catalysts to improve oil yield in HTL treatment was studied by Ross *et al*. (2010). They evaluated the influences of temperature and catalyst type (alkali, potassium hydroxide and sodium carbonate and the organic acids, acetic acid and formic acid) on the production and nature of biocrude produced by *Chlorella* and *Spirulina* after HTL. Their results show that biocrude yield is higher in the presence of organic acids and at higher temperatures. Due to the high amounts of nitrogen in chlorophyll and proteins in algal cells, the process may lead to high NO_x_ emissions, one of the biggest bottlenecks for this process to be a real alternative to biofuel production (Barreiro *et al*., 2013). Garcia Alba *et al*. (2011) found that hydrothermal treatment of *Desmodesmus* sp. produces up to 6% of nitrogen content in the oil yield, while Toor *et al*. (2013) reported a nitrogen content of 4–6% in oil extracted from *Chlorella* and *Spirulina*. Scalability, safety, and equipment costs and maintenance appear to be issues that deserve further investigation.

### Osmotic shock

By osmotic shock, algae cells burst, liberating their contents due to an abrupt lowering of osmotic pressure (Mercer and Armenta, 2011). Lipid extraction after osmotic shock has been studied during recent years (Lee *et al*., 2010; Prabakaran and Ravindran, 2011; Yoo *et al*., 2012). Yoo *et al*. (2012) evaluated lipid recovery from wet biomass of *Chlamydomonas reinhardtii* by osmotic shock along with both polar and non‐polar organic solvents. Their results suggest that osmotic shock could increase lipid recovery approximately two times. Despite being a very simple method for cell disruption, osmotic shock is not widely employed as it depends highly on cell wall properties; a higher lipid recovery could be achieved with other methods, such as microwave extraction or sonication (Lee *et al*., 2010; Prabakaran and Ravindran, 2011). These methods can be applied in the same way to different species, while the osmotic shock procedure is species‐dependent (Yoo *et al*., 2012). However, if well developed for one species, an efficient osmotic shock pre‐treatment can be highly desirable as it is scalable and does not require any special equipment.

### Enzymatic disruption

Cell disruption using enzymes is an alternative for lipid extraction that has been poorly studied for algal cells. Enzymatic treatment results in a good lipid recovery with the advantage of disrupting cells with minimal damage to the target product due to high selectivity of the reactions (Mercer and Armenta, 2011; Demuez *et al*., 2015). A successful oil extraction from plant seeds was reported by Shah *et al*. (2004) with a combination of sonication and enzyme treatment, while 95% of oil was recovered from borage seeds with enzymatic treatment under cold pressing conditions (Soto *et al*., 2007). In microalgae, enzymatic hydrolysis with immobilized cellulose was studied by Fu *et al*. (2010) to break cell walls of *Chlorella* sp. resulting in a lipid extraction efficiency of 56% (14% more than unhydrolysed microalgae). Likewise, the enzymatic hydrolysis with cellulase on *C. vulgaris* cultures enhanced lipid extraction by 1.73‐fold compared with unhydrolysed cultures (Cho *et al*., 2013). The results of Zheng *et al*. (2011) show that enzymatic treatment on *C. vulgaris* had a lipid recovery of 7%, 22% and 24% with snailase, lysozyme and cellulose respectively, while Taher *et al*. (2014) reported the highest extraction yield of 16.6% using lysozyme. Liang *et al*. (2012) achieved the highest lipid recovery (around 35%) with snailase and trypsin in comparison to cellulase (16%), neutral protease (12%) and alkaline protease (8%). Besides, several studies of enzymatic hydrolysis in microalgae to enhance bioethanol (Choi *et al*., 2010; Rodrigues and Bon, 2011; Kim *et al*., 2014) and biogas production (Ciudad *et al*., 2014; Mahdy *et al*., 2014; Ometto *et al*., 2014) have reported the efficiency of this method.

### Oxidative stress

Oxidative stress on algal cells has been recently studied by Bai *et al*. (2014) by applying different concentrations of free nitrous acid (FNA) as pre‐treatment for oil extraction in order to enhance the extraction efficiency. The authors report a lipid yield 2.4‐fold higher for cultures treated with FNA (up to 2.19 mg HNO_2_‐N/L). This is a promising technique that requires further studies as FNA is considered a green and renewable chemical (Wang *et al*., 2013) that may lower production costs. Other oxidative agents or UV light have also been proposed as a suitable pre‐treatment method to improve lipid extraction efficiency (Sharma *et al*., 2014).

### Electroporation

Electroporation of wet algal biomass can be induced by applying a pulsed electric field to the cells, creating aqueous pores in the cell walls, enhancing mass transfer across the cell membrane. It is currently well established in molecular biology, by which electroporation can be used to facilitate the transportation of drugs, chemicals and foreign DNA products into the cell (Ho and Mittal, 1996); however, there have been very few studies conducted as to whether it is an efficient method for lipid extraction in microalgae. Sommerfeld *et al*. (2008) determined that electroporation achieved a total lipid extraction of 92% from *Pseudochlorococcum* sp. in comparison to 62% using the standard Bligh and Dyer method.

### Supercritical carbon dioxide extraction

The traditional use of organic solvents for lipid extraction could be displaced by supercritical carbon dioxide (SCCO_2_) as an alternative solvent. SCCO_2_ is a green technology which is also efficient at extracting TAG and other lipid components, while it has a lower toxicity and produces an organic solvent‐free extract in a shorter extraction time compared with the use of organic solvents (Andrich *et al*., 2005; Halim *et al*., 2011; Soh and Zimmerman, 2011). Halim *et al*. (2011) compared SCCO_2_ extraction with hexane extraction of lipids from the marine microalga *Chlorococcum* sp. concluding that hexane extraction is significantly less efficient as it required about 5 times longer to achieve a comparable lipid yield in comparison to SCCO_2_. However this technique is suffering from the high costs associated with its energy consumption, required infrastructure and operation (Halim *et al*., 2011).

## Future directions

**Figure 2:**
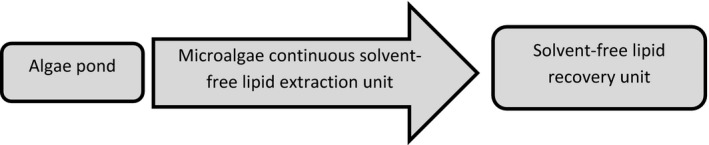
Proposed process for solvent‐free lipid extraction and recovery from wet, concentrated microalgae.

**Figure 3:**
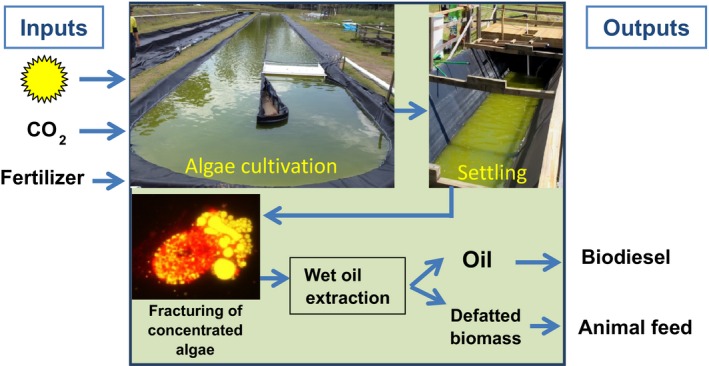
Example of a scenario for industrial production and extraction of microalgal lipids. Shown are the Algae Energy Farm at the University of Queensland with algae cultivation in raceway ponds, harvesting of microalgae by settling in a vee‐shaped pond, followed by mechanical fracturing of microalgal cells in concentrated algal slurry. The freed‐up oil droplets (shown in yellow by Nile red staining) and the remaining biomass (red) are then separated into oil and defatted biomass, which among many other applications, can be used for biodiesel and feed production respectively. Further optimization steps are required for the efficient separation of oil and defatted biomass.

When considering different extraction techniques, the main consideration should be placed on costs, scalability, safety and environmental concerns (Table 2). To make microalgal lipid extraction/recovery a more economically viable option which can compete better with conventional oil industries, there is a need to introduce and develop techniques which are not only efficient but also safe to operate, safe to the environment and sustainable. Hence, a major aim for future work could be the development of safe and low‐cost mechanical (solvent‐free) lipid recovery technology (Fig. 2). A desirable solvent‐free technique is one which can be applied *in situ* and at large scale, making microalgal lipid extraction/recovery a continuous process directly linked to algae cultivation. A promising approach seems to be the use of mechanical rupturing without the use of organic solvents (Fig. 3). Most economical and environmentally friendly could be the use of thermal or osmotic shock pre‐treatments which, depending on the cell wall properties of the microalgae, can result in the release of lipid bodies in the surrounding liquid. Next, oil droplets from the oil‐in‐water emulsion then need to be recovered. While organic solvents are suitable for this process, other mechanical separation technologies (e.g. by ultrafiltration) can be applied. Clearly, this area deserves further development. The co‐production of microalgal oil and protein‐rich biomass for the production of biodiesel and animal feed, respectively, has been discussed as a biorefinery concept in the literature. Both products are essentially produced at the same cost as one cannot be produced without the other. However, economical feasibility has yet to be established for these two low‐value products. Therefore, the industry has focussed more on higher value products, such as high‐protein microalgal biomass, omega‐3‐rich microalgal oil and microalgae‐derived carotenoids. Recent techno‐economic analyses have indicated that with the use of a cheap source of CO_2_ ($40/ton), microalgal oil and biomass can be produced for as little as $2250/kL (Davis *et al*., 2011) and $1790/ton (Slade and Bauen, 2013), respectively, but data from actual large‐scale production demonstration farms have yet to be included.

**Table 2:** Comparison of different lipid extraction techniques from microalgae

Method	Safe and environmentally friendly	Easily scalable	Economical	Efficient on wet biomass	Purity of the final product	Efficiency	Fast operational time	Other advantages/limitations
Ultrasound‐assisted extraction	✓	□	✗	✓	✓	✓	✓	Low set‐up costs Reducing thermal denaturation of essential biomolecules Possible oxidation of lipids due to prolonged exposure
Microwave‐assisted extraction	✓	✗	✓	✓			✓	Inefficient on non‐polar or volatile compounds and/or the solvent(s) High maintenance costs at commercial scale
Hydrothermal liquefaction	✗	✗		✓	✗	✓	✓	Applicable on wet biomass with water content as high as 80–95% Can achieve an energy recovery from biomass to fuel up to 80% Process may lead to high NO_x_ emissions High equipment and maintenance costs
Enzymatic disruption	✓	✓	✗	✓	✓	✓	✗	Minimal damage to the target product Long process duration Species‐dependent
Supercritical carbon dioxide extraction	✓	✗	✗	✗	✓	✓	✓	Green technology Uncontaminated products
Electroporation	✓	✓	✓	✓				Insufficient studies on its application for microalgal lipid extraction
Oxidative stress	✓	✓	✓	✓	✓	✓	✗	Long process duration
Use of combination solvents	✗	✗	✗	✓	✗	✓	✓	
Osmotic shock	✓	✓	✓	✓	✓			No special equipment required Depends highly on cell wall properties Species‐dependent

To make co‐production of microalgal oil for biodiesel and biomass for feed a reality, it is essential that further cost reductions are applied to all steps. This should also include the concentration of microalgal cultures to algal slurries. The most cost‐effective way is probably settling (gravity‐assisted sedimentation; Fig. 3), while water can be recycled for further cultivation. Further cost savings can be achieved with large‐scale extraction, and the required process optimizations for wet extraction at large scale may bring further benefits. Importantly, the use of the remaining biomass after lipid extraction should be considered. Additional lipids may be recoverable from the partially defatted algal biomass by using organic solvents, as is common practice with feedstocks for vegetable oil. However, the use of organic solvents can result in residues remaining in the defatted algal biomass, therefore a solvent‐free extraction process would be preferable if the remaining biomass is to be used for human consumption or animal feed.
